# Evaluation of Companion Diagnostics in Scientific Advice and Drug Marketing Authorization Applications by the European Medicines Agency

**DOI:** 10.3389/fmed.2022.893028

**Published:** 2022-05-06

**Authors:** Marc Maliepaard, Priscilla Nibi, Gabrièlla Nibi, Anna M. G. Pasmooij

**Affiliations:** ^1^Dutch Medicines Evaluation Board (CBG-MEB), Utrecht, Netherlands; ^2^Department of Pharmacology and Toxicology, Radboud University Medical Centre, Nijmegen, Netherlands

**Keywords:** companion diagnostics (CDx), *in vitro* diagnostic (IVD), EU regulation 2017/746, European Medicines Agency (EMA), regulatory science, drug marketing authorization applications, scientific advice

## Abstract

With the implementation of the new EU regulation on *in vitro* diagnostics (IVDR) in May 2022, notified bodies will be required to assess Companion Diagnostics (CDx). The EMA and national medicines agencies will be consulted on the performance and safety of CDx. In this paper, we report on our systematic review on how the EMA has dealt with CDx in dossiers for marketing authorization procedures, in 2017–2019, and in scientific advice procedures in 2016–2020, prior to the implementation of the new IVDR. Out of 167 medicines approved or refused by the EMA, CDx played a role for 20 medicines during assessment. Both European public assessment reports (EPARs) and the internal day 80 and day 120 assessment reports (ARs) of the EMA centralized marketing authorization procedures for these 20 medicines were analyzed in detail to determine how CDx were assessed. Likewise, in 46 of 159 cases in which scientific advice was provided, CDx were mentioned in the question-and-answer section of the scientific advice, and these were analyzed in an analogous manner. Our analysis indicates that clinical performance and analytical performance of the CDx were the most-discussed topics, being discussed 11 and seven times in the 20 EPARs and 59 and 29 times in the ARs, respectively. For scientific advice, clinical and analytical performance was discussed 65 and 22 times in the 46 retrieved mentions of scientific advice. Other aspects in relation to CDx were discussed as well, although at a lower frequency, in assessment reports and scientific advice. Overall, our analysis demonstrates that, despite the absence of an obligation from a legal point of view, EMA has gained experience on the assessment of CDx, most notably regarding its analytical and clinical performance. This experience may be useful in situations in which the EMA and national agencies of EU member states will formally be consulted by notified bodies regarding the performance and safety of CDx. In addition, the issues raised in the EPARs, ARs and scientific advice reports provide insight for applicants on aspects of CDx that need careful consideration.

## Introduction

Globally, the healthcare system is transitioning from a “one-size-fits-all” approach to a precision medicine approach. It has become increasingly clear that not all medicines are suitable for every patient, and, therefore, selection of the right drug for a patient is crucial. For this purpose, a subset of *in vitro* diagnostics (IVDs) that are already indispensable in precision medicine are the companion diagnostics (CDx). A CDx is a validated test for a predictive biomarker (PBM), which enables the identification of subjects at a higher risk of developing adverse reactions to the medicine in question (safety) or the identification of a subset of patients who have an increased chance of efficacy (1, 2). Testing with an adequate CDx is a prerequisite to the successful and/or safe use of the corresponding medicine and is essential before the initiation of treatment. A prime example of the need for a CDx is trastuzumab, which is registered for use in the treatment of human epidermal growth factor receptor 2-positive (HER2+) breast cancer already since the year 2000. Since trastuzumab is effective only in the HER2+ subset of patients, a CDx is required to determine whether the tumors of a particular patient are HER2+ (3).

**Figure 1:**
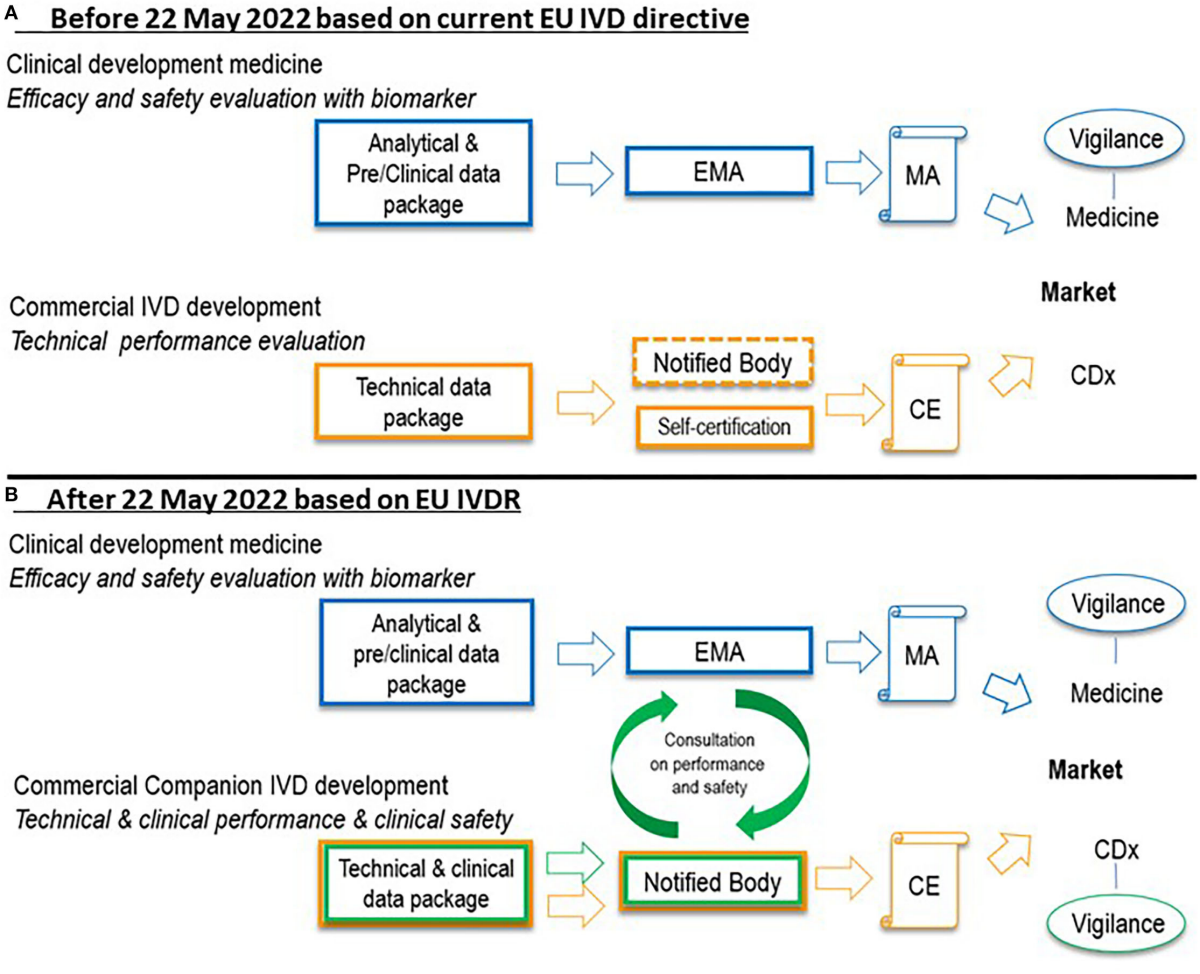
Situation before 22 May 2022 **(A)** and after 22 May 2022 **(B)** with regard to certification of a new CDx in combination with a medicine in the European Union. In the current situation under the IVD directive, technical data in support of CDx are not assessed by notified bodies (self-certification), indicated by the dashed notified body box line in **(A)**. Under the IVDR after 22 May 2022, such data, as well as performance data, will be assessed by notified bodies before granting a CE mark. EMA or national agencies of EU member states will be consulted by the notified body on performance and safety of the CDx. Further, a vigilance system will be established for CDx. Clinical data in support of medicine registration in blue, technical data in support of CDx certification in orange, performance and safety data in support of CDx certification in green. CDx, companion diagnostic; CE, Conformitè Europëene mark; EMA, European Medicines Agency; EU, European Union; IVD, *in vitro* diagnostic; IVDR, *in vitro* diagnostic regulation 2017/746; MA, marketing authorization.

Until 2017, CDx were not defined in the European legislation and, as a consequence, manufacturers were allowed to self-certify CDx to obtain a Conformitè Europëene (CE) mark. A CE mark indicates that the product may be sold freely in any part of the European Economic Area. In the current situation, scientific data supporting the quality and performance of CDx are not assessed by the notified bodies. The current situation with respect to certification of CDx is shown in Figure 1A.

However, in May 2017, the term CDx was introduced in the EU regulation on IVDs, Regulation (EU) 2017/746. New CDx should follow the IVDR from May 2022 (4). A progressive roll-out of the IVDR will be employed; For CDx already CE-marked under the current EU IVD directive, denoted ‘legacy IVD’ in the IVDR, a transition period until May 2025 is in place, after which date these products should comply with the new IVDR (5). The new EU IVD regulation reveals two significant changes to the current situation. Firstly, CDx will be classified as having a high individual risk or a moderate public risk (category C) (1). For IVDs in this category, proof of clinical conformity of an IVD, which will be assessed by notified bodies, is now requested prior to being granted marketing authorization. Secondly, the European Medicines Agency (EMA) and national medicine agencies will also contribute to the assessment of CDx. More specifically, the medicine agencies in the EU will be consulted by the notified bodies with regard to the performance and safety of CDx (see Figure 1B). With the changing role of the notified bodies in the assessment of CDx, the role of the EMA changes as well, and the EMA will have to prepare for this new role.

Importantly, differences exist between CDx development in Europe and the United States. First, in the United States, the FDA do the complete assessment of the CDx, as a cooperation between Center for Devices and Radiological Health and Center for Drug Evaluation and Research/Center for Biologics Evaluation and Research, without involvement of private notified bodies (6, 7). For this reason, in case a CDx is co-developed together with a new medicine, the medicine and the CDx will normally be approved at the same time by FDA. By contrast, according to the European legislation, a CDx for a certain PBM is approved by a notified body, separately from the registration of the accompanying medicine by EMA or national medicines agencies (8). Further, in the United States, the term “complementary” diagnostics is used, which means that although a certain biomarker is determined, this type of test is optional rather than mandatory before the use of a certain medicine (8). However, this terminology is so far not part of the US legislation. Also, in the EU, complementary diagnostics are not formally defined.

To prepare for this new role and with the new EU regulations in mind, the EMA released a concept paper in 2017 on PBM-based assay development in the context of drug development and life cycle (9). This concept paper is intended to be the first step in the preparation of a guideline that will address the developmental challenges of precision medicine with CDx, following implementation of the new IVD regulation in 2022.

Over the past couple of years, the EMA and national agencies in EU member states have received information on CDx in applications for marketing authorization for new medicines as well as for scientific advice. Recently, it was reported that, for a number of medicines registered in the EU, information on CDx is provided in the summary of product characteristics (SmPCs) and in the European public assessment report (EPAR) (10). The EPAR is a set of documents describing the evaluation of a medicine authorized *via* the centralized procedure and includes the product information. The EPAR is published on the EMA website. Our focus for such products was on the assessment of the CDx by EMA. More insight into these previous assessments and the scientific advice regarding CDx by the EMA could be useful for finalization of the future EMA CDx guideline in support of the implementation of the new EU IVD regulation. Therefore, we have investigated whether and how the EMA has assessed CDx present in dossiers for new medicines in 2017–2019. Secondly, we have determined the advice given regarding CDx in EMA scientific advice reports in 2016–2020.

## Materials and Methods

### Selection and Analysis of Marketing Authorization Dossiers for Medicines With Companion Diagnostics Involved

Medicines approved or refused in January 2017–December 2019 were selected from a table of EPARs for all human medicines in 1995–2020. These were downloaded from the EMA website (https://www.ema.europa.eu/en). Of these medicines, biosimilar and generic medicines were excluded, because any relevant information on CDx that these medicine dossiers might contain would be found in the EPAR and the discussion of the original medicine.

The SmPCs of the selected medicines were screened for the presence of IVD or diagnostic test-related information in Sections 4.1 (indication), 4.2 (posology), and 4.3 (contra-indications). Mentioning of IVD or diagnostic test-related information in section 4.4 (Special warnings and precautions for use) was not analyzed, since it was considered that the use of an IVD, when indicated in that section, is not obligatory. When an IVD or diagnostic test was mentioned in one of the SmPC Sections 4.1, 4.2 or 4.3, the test was considered to be a potential CDx. In case a potential CDx was indicated in the SmPC, the EPARs for the medicines involved were screened for any further mention of a specific CDx and for further discussions concerning this CDx. This step led to the final identification of all medicines with a discussion of CDx. For the medicines for which a CDx was discussed in the EPAR, the EPARs were systemically checked for information on the following four topics: 1) analytical performance of the CDx; 2) clinical performance of the CDx; 3) interchangeability of CDx assays, including concordance testing and bridging studies and tests for the same PBM; and 4) testing of stored or fresh patient samples, the latter encompassing both the appropriate preservation of the sample, e.g., fresh, frozen, or various fixations, as well the liquid biopsy or tumor biopsy. These four categories were chosen based on the EMA concept paper on predictive biomarker-based assay development in the context of drug development and life cycle (9).

Furthermore, for medicines for which a CDx was identified and discussed in the EPAR, the internal EMA assessment reports (ARs) of the centralized procedures—specifically, the day 80 (first assessment round) ARs of the rapporteur and co-rapporteur and the questions raised in the joint day 120 AR—were screened for information on the assessment of the CDx during the marketing authorization procedures. Statements and questions found in the ARs and the EPARs concerning the CDx were collected. In case a specific statement or question was mentioned multiple times within the documents for the same product, it was only noted once, since this would not lead to new information about the CDx. Information on the CDx in the EPAR and ARs were collected separately.

The statements found in the EPAR and ARs were assigned to the four categories as indicated above. Statements about the CDx that were considered relevant but that could not be assigned to one of these categories were also noted and categorized as “other.” Subsequently, the results for the four topics were compared to identify the ranking order in which they were discussed. Further, the results from the different sources (EPARs and ARs) were compared to identify whether any differences were noticeable between the statements on PBMs and CDx in these two data sources. Of note, due to confidentiality of the internal ARs, the statements from the ARs were reported only in the subcategories and are not cited in this paper.

### Selection and Analysis of Scientific Advice for Medicines With Companion Diagnostics Involved

Potentially relevant scientific advice reports provided in January 2016–July 2020 were collected from the EMA Scientific Advice Working Party (SAWP) database, using a search tool and keywords in the EMA SAWP system. Access to the internal EMA SAWP database was granted to MM and MP due to their appointment at the Dutch Medicines Evaluation Board. The following keywords and strings were used: “*companion AND diagnostic AND performance,” “companion AND diagnostic OR companion AND diagnostics,” “in vitro diagnostic,” “validated test,”* and “*predictive biomarker AND in vitro.”*

After removing duplicate scientific advice, to identify the relevant advice, a text search was conducted in each of the scientific advice reports found in the SAWP database, *using “CDx,” “biomarker,” “companion,” “diagnostic,” “in vitro,” and “validated test.”* Text surrounding the keyword hit was checked for relevance regarding CDx. Scientific advice reports with CDx discussed in the Q&A section were considered relevant, whereas scientific advice with information on CDx only in the introduction or background information included in the advice were discarded.

For the scientific advice that was finally selected, questions asked by companies regarding CDx and the matching advice given by the SAWP were analyzed and structured based on the same four categories: 1) analytical performance of the CDx; 2) clinical performance of the CDx; 3) interchangeability of CDx assays, including concordance testing and bridging studies and tests for the same PBM; and 4) testing of stored or fresh patient samples. The total number of mentions of CDx per category were compared. Within each category, subgroups were formed based on frequently reoccurring CDx-related topics seen in the discussions, and answers were sorted according to these subgroups. Answers were labeled as “other” when they did not fit into one of the four categories.

Of note, due to confidentiality of the scientific advice, examples of advice-related questions provided in this publication are done so in a descriptive and anonymized manner only.

## Results

### Selection of Medicines From Marketing Authorization Dossiers for Further Evaluation

**Figure 2:**
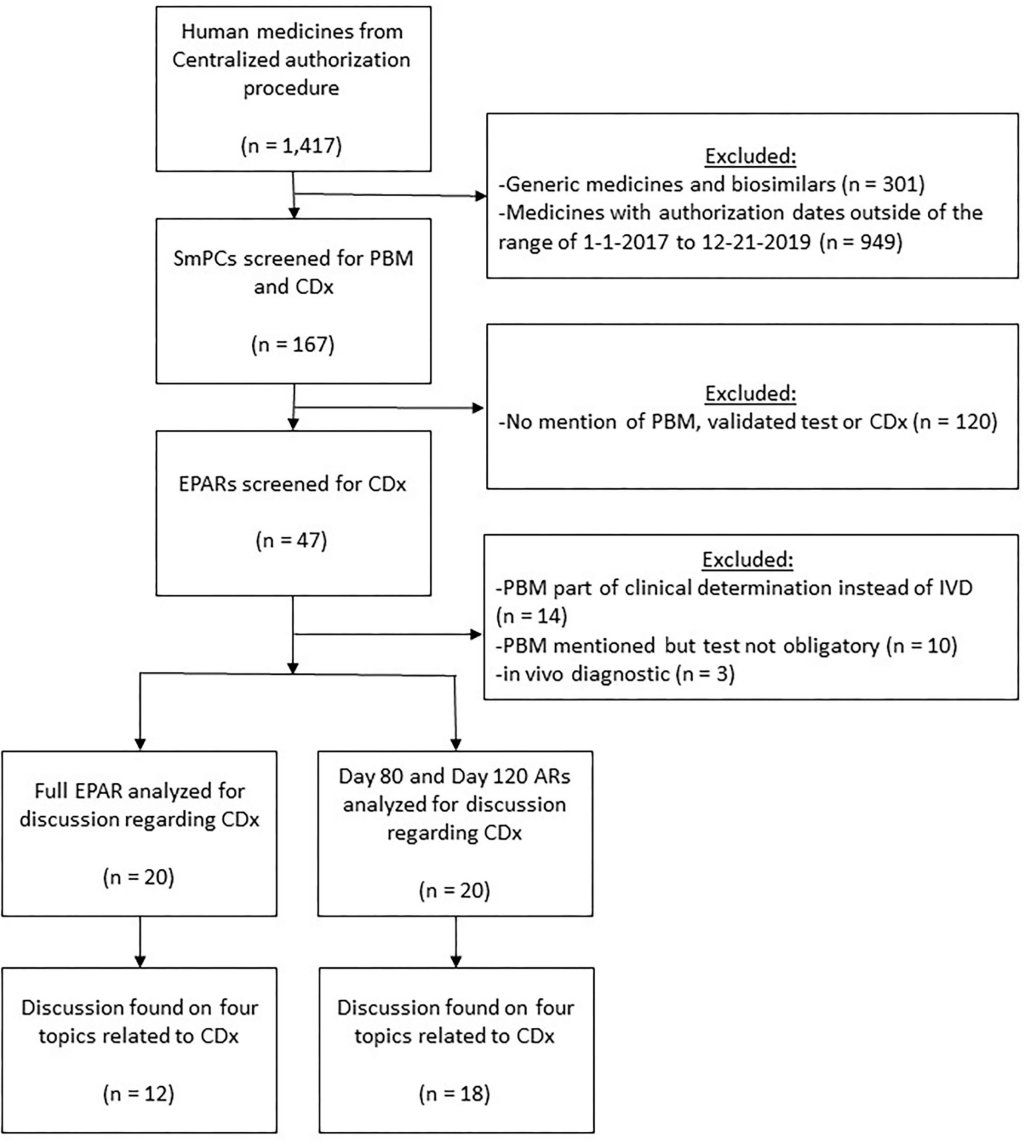
Flowchart visualizing the selection of medicines with CDx involved that were registered by EMA in January 2017–December 2019. AR, assessment report; CDx, companion diagnostics; EPAR, European public assessment report; PBM, predictive biomarker; Q&A, question and answer; SA, scientific advice; SmPC, summary of product characteristics.

First, a table containing all (1,417) registered or refused human medicines in 1995–2020 was downloaded from the EMA website on February 17, 2020. Exclusion of biosimilar and generic medicines, as well as medicines with marketing authorization or refusal dates outside of January 2017–December 2019 resulted in the elimination of 167 medicines (Figure 2). Subsequently, screening of the SmPCs to assess whether any warnings or advice were provided concerning a PBM, validated test, or CDx for that specific medicine identified 47 medicines for which a CDx was potentially included. More thorough examinations of the EPARs of these 47 medicines resulted in 20 medicines with information on a CDx. For these 20 products, both the EPAR and the internal AR were analyzed in detail for discussion of CDx assessment. The 27 medicines that were excluded at this stage concerned medicines for which the biomarker does not directly affect the efficacy or safety of the medicine. Excluded cases in this step were those in which the PBM was used in determining the clinical diagnosis, however, the PBM was not a target for the medicine. Although the high quality requirements as posed on CDx should ideally also apply for assays toward such PBM, we decided to exclude these cases, since in the new IVDR the term CDx only refers to a device which is essential for the safe and effective use of a corresponding medicine. This implies that in cases where the PBM assay is not directly related to a medicine, but opens the way for potentially multiple accepted treatment modalities for a certain indication, it is not considered a CDx. Further, excluded cases concerned medicines for which PBM testing is not obligatory (Specific reasons for excluding these medicines are provided in Supplementary Table 1).

Information about the 20 selected medicines is provided in Table 1. Seventeen of the 20 medicines (85%) were antineoplastic agents (anatomical therapeutic chemical [ATC] code L). Further, one medicine (5%) was a nervous system medicine (ATC code N), sensory organs medicine (ATC code S), or respiratory system medicine (ATC code R).

**Table 1:** Discussion of CDx analytical performance, clinical performance, concordance and interchangeability, and testing of stored vs. fresh patient samples in the EPARs and ARs of the 20 selected medicines.

**International non-proprietary name (INN)**	**EU medicine brand name**	**Biomarker**	**CDx-related topic**							
			**CDx analytical performance**		**CDx clinical performance**		**Concordance and interchangeability**		**Testing stored vs. fresh patient samples**	
			**EPAR**	**AR**	**EPAR**	**AR**	**EPAR**	**AR**	**EPAR**	**AR**
alectinib	Alecensa	ALK	–	–	1	4	–	–	–	–
brigatinib	Alunbrig	ALK	–	–	–	1	–	1	–	–
lorlatinib	Lorviqua	ALK	–	1	1	3	–	–	–	2
binimetinib	Mektovi	BRAF V600 mutation	–	–	1	2	–	1	–	–
encorafenib	Braftovi	BRAF V600 mutation	–	–	–	1	–	–	–	–
rucaparib	Rubraca	BRCA1/2 mutations	1	3	–	1	2	3	1	1
talazoparib	Talzenna	BRCA1/2 mutations	–	1	1	7	1	1	–	–
dacomitinib	Vizimpro	EGFR activating mutations	–	2	–	2	–	2	–	–
gilteritinib	Xospata	FLT3 mutation	–	–	1	4	–	2	–	–
midostaurin	Rydapt	FLT3 mutation	1	2	1	4	–	2	–	–
atezolizumab	Tecentriq	PD-L1	1	4	1	8	–	1	–	–
durvalumab	Imfinzi	PD-L1	1	2	1	6	–	1	–	–
inotuzumab ozogamicin	Besponsa	CD22	2	2	–	3	2	3	–	–
larotrectinib	Vitrakvi	NTRK gene fusion	1	9	2	6	1	1	2	–
abemaciclib	Verzenios	HER2 negative	–	–	–	–	–	–	–	–
neratinib	Nerlynx	HER2 positive	–	–	–	–	–	–	–	–
ribociclib	Kisqali	HER2 negative	–	3	–	1	–	–	–	–
brexpiprazole	Rxulti	CYP2D6 PM	–	–	1	1	–	–	–	–
voretigene neparvovec	Luxturna	RPE65 mutations	–	–	–	3	–	1	–	–
tezacaftor/ivacaftor	Symkevi	homozygous for the F508del mutation or heterozygous + one of the mutations mentioned in the SmPC	–	–	–	2	–	–	–	–
Total			7	29	11	59	6	19	3	3

*The number in each cell represents the number of occasions a specific topic was mentioned*.

*ALK, anaplastic lymphoma kinase; AR, assessment report; BCRA, breast cancer gene; BM, biomarker; BRAF, B-raf proto-oncogene; CD22, cluster of differentiation 22; CDx, companion diagnostic; EGFR, epidermal growth factor receptor; EPAR, European public assessment report; FLT3, fms-like tyrosine kinase 3; HER, human epidermal growth factor receptor; HR, hormone receptor; NRTK, neurotrophic tyrosine kinase receptor; PD-L1, programmed death-ligand 1; RPE65, retinal pigment epithelium-specific 65 kDa protein*.

### Selection of Medicines From Scientific Advice Reports for Further Evaluation

**Figure 3:**
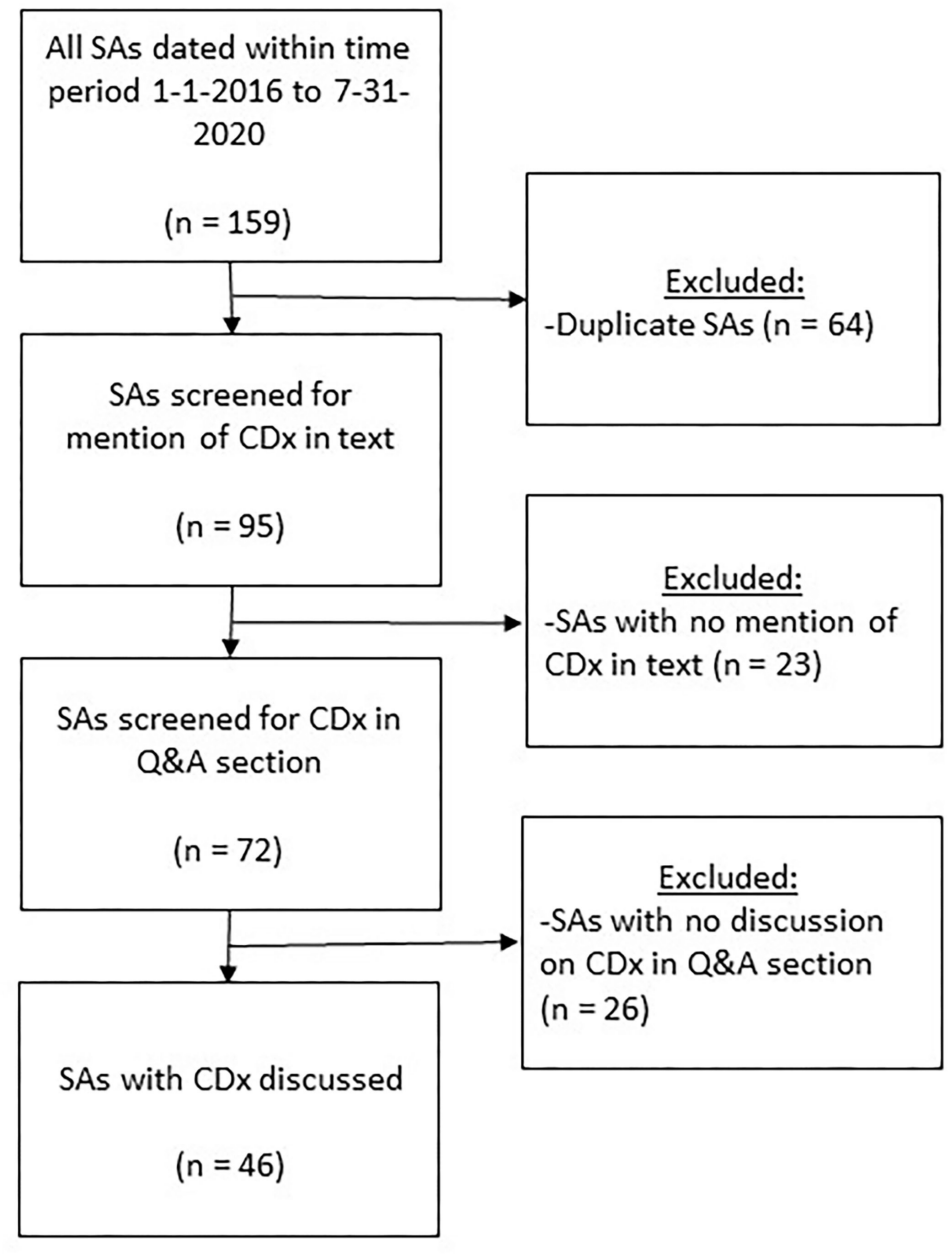
Flowchart visualizing the selection of medicines with CDx involved, and discussed in the question-and-answer section in scientific advice reports provided by the EMA in January 2016–July 2020. CDx, companion diagnostics; Q&A, question and answer; SA, scientific advice.

For the time period January 2016–July 2020, a total of 159 potentially relevant scientific advice reports were found in the EMA SAWP database applying the keyword search strategy in August 2020 (Figure 3). After removing the duplicate scientific advice obtained by the overlapping text searches, 95 scientific advice reports remained. From these 95 advice reports retrieved by using “*CDx,” “biomarker,” “companion,” “diagnostic,” “in vitro,”* and “*validated test”* text searches, 72 were found with a CDx mentioned in the advice. Manual screening of each of these advice reports yielded 46 with a CDx specifically mentioned in the question-and-answer section, specifically, 16 from 2016, seven from 2017, 11 from 2018, 10 from 2019, and two from January–July 2020. Most medicines were antineoplastic agents (42 of 46, 92%, ATC code L). Further, one of 46 (2%) was a blood and blood-forming organs medicine (ATC code B), cardiovascular medicine (ATC code C), dermatological medicine (ATC code D), or musculoskeletal system medicine (ATC code M).

### Companion Diagnostics-Related Discussion in European Public Assessment Reports and Assessment Reports

For the 20 selected medicines, mentions of CDx analytical performance, clinical performance, testing of stored patient samples vs. fresh samples, and interchangeability including concordance testing and bridging studies in the EPARs or the day 80 and day 120 ARs are summarized in Table 1.

With respect to the EPARs, one of the four topics was mentioned at least once for 12 of the 20 selected medicines, and for seven of the 20 medicines, more than one topic was mentioned. CDx-related topics were mentioned a total of 27 times. Most of the CDx-related discussions in the EPARs (11) focused on the clinical performance of the CDx, while seven highlighted the analytical performance, six the concordance testing and bridging, and three the testing of stored vs. fresh patient samples. For eight of the 20 medicines, no specific mention was made about these CDx-related topics in the EPARs.

With respect to the ARs on day 80 and day 120, one of the four topics was mentioned at least once for 18 of the 20 selected medicines, and for 12 of the 18 medicines, more than one topic was discussed in the ARs. CDx-related discussions were mentioned, in total, 110 times for these 18 medicines. Most CDx-related discussions in the ARs (59) focused on the clinical performance of the CDx, 29 on the analytical performance, 19 on the concordance testing and bridging, and three on testing of stored vs. fresh patient samples. For two of the 20 medicines, no specific mention was made about these CDx-related topics in the EPARs.

Details on the remarks regarding CDx that were noted in the EPARs are included in Supplementary Table 2. In addition, a number of examples are provided below.

With respect to analytical validity, remarks are included in the EPARs indicating that, at a certain point during assessment, discussion on the CDx had taken place: “*The immuno-assays for quantification of durvalumab and soluble PD-L1 in human serum samples were adequately validated*” (11), “*The VENTANA PD-L1 (SP142) immunohistochemistry assay was not validated for intended use to measure PD-L1 expression on tumor cells in urothelial carcinoma.”* (12), and “*The validation report and addendums regarding peripheral blood specimens and inter-laboratory qualification were provided”* (13).

With respect to clinical performance, a number of discussions in the EPARs were summarized in questions about the suitability of the CDx to accurately predict the disease state *via* the PBM: “*PD-L1 expression proved to be rather of prognostic than of predictive value in this data set.”* (11), and “*In addition, genomic analysis of baseline samples remaining after centralized BRAF testing would be informative to assess whether there is a relationship between baseline mutations and efficacy outcomes.”* (14). Additionally, the cut-off of a CDx was discussed, for example, for durvalumab: “*it is not entirely clear how and why the cut-off is at 25%”* (10). The applicant was asked to provide additional subgroup analyses. Furthermore, the need for the CDx, whether as a companion or complementary diagnostic, was raised as an issue: “*The necessity of a mandatory genotyping assay should be discussed by the applicant”* (15).

Discussion of the interchangeability of assays was noted in the EPARs when multiple methods of PBM determination were available in the research setting and no real golden standard had been established yet. This was expressed through remarks such as, “*There are methods available in a research setting, to test for BRCA1/2 locus-specific loss of heterozygosity; however, the Scientific Advisory Group could not confirm to what extent any particular test is well-established”* (16). Additionally, the topic of concordance between local testing and central testing was mentioned. An example was, “*there was a high positive agreement between local and central BRCA results”* (17). Furthermore, the question of whether the use of liquid biopsies would be as reliable as tumor samples for PBM measurements was raised on three occasions.

With respect to testing of stored vs. fresh patient samples, the issue toward the appropriate preservation of the sample, e.g., fresh, frozen, or various fixations, was discussed. Furthermore, the question whether the use of liquid biopsies would be as reliable as tumor samples for predictive biomarker measurements was raised several times. In addition to liquid biopsies, tumor samples were often requested and subsequently tested. In the larotectinib EPAR (18), the following was stated by the Scientific Advisory Group, “*Liquid biopsies should be further investigated; these should be performed with a joint tumour biopsy up-front and ideally at cancer progression”*.

**Figure 4:**
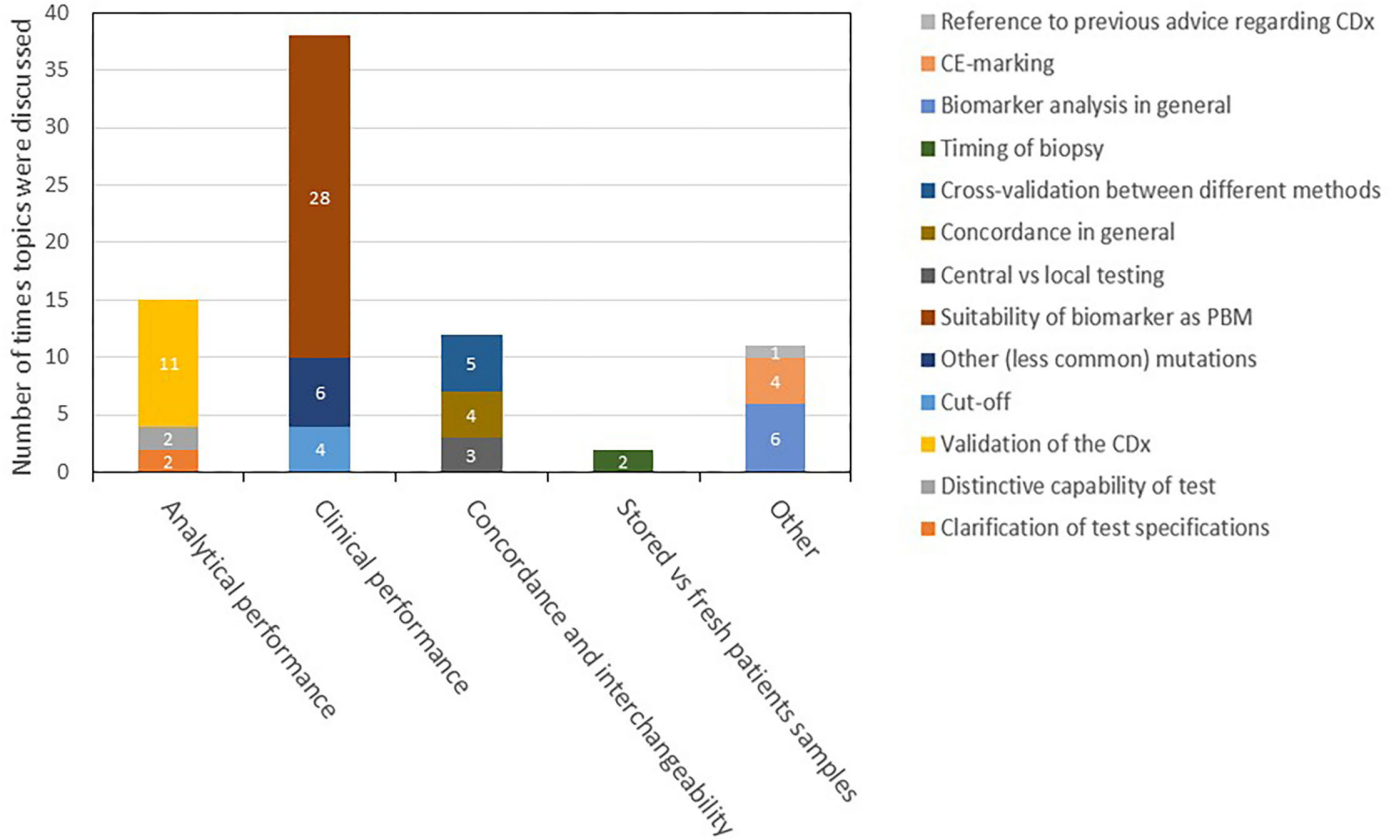
Description and categorization of remarks on CDx found in *day 80 ARs* for medicines registered in 2017–2019. The four topics of CDx analytical performance, clinical performance, concordance and interchangeability, and testing of stored vs. fresh patient samples were further divided into subtopics. Remarks that did not fit to any of the four topics were categorized as “other.” AR, assessment report; CDx, companion diagnostic; EPAR, European public assessment report; PBM, predictive biomarker.

**Figure 5:**
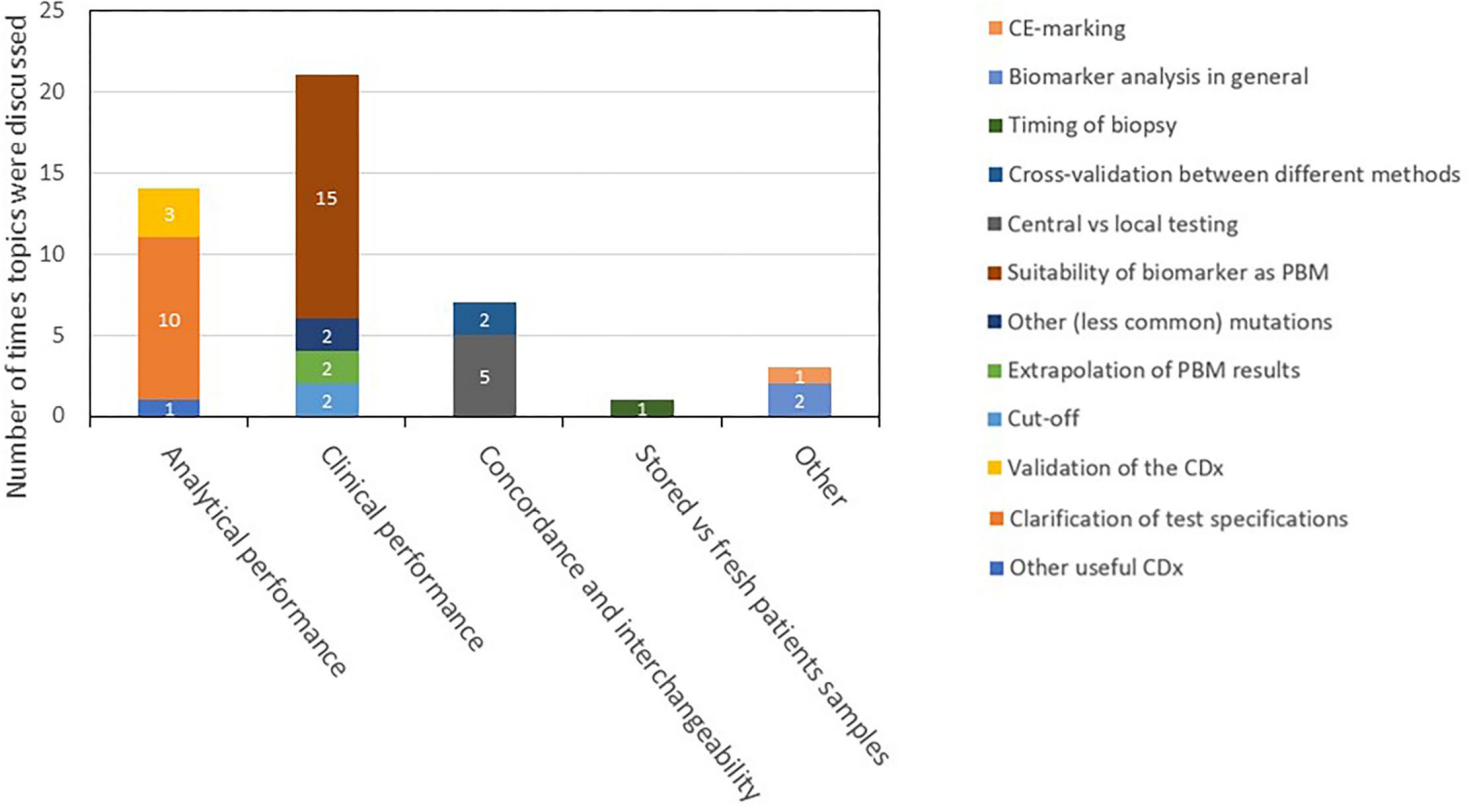
Description and categorization of CDx found in the *day 120 ARs*. The four topics of CDx analytical performance, clinical performance, concordance and interchangeability, and testing of stored vs. fresh patient samples were further divided into subtopics. Remarks that did not fit to any of the four topics were categorized as “other.” AR, assessment report; CDx, companion diagnostic; EPAR, European public assessment report; PBM, predictive biomarker.

With respect to the ARs, the same issues in general were mentioned as in the EPARs, though in a more elaborate fashion. Further, besides the four topics of CDx analytical performance, clinical performance, testing of stored patient samples vs. fresh samples, and interchangeability, there were remarks about CDx or PBM in the ARs that could not be assigned to one of the prespecified specific topics because they were about biomarker assays in general. These remarks were labeled as “other.” Since the ARs are not public, it is not possible to present the same detail as for the EPAR assessment in this report. However, the findings in the day 80 and day 120 ARs are summarized in a descriptive manner in Figures 4, 5, respectively.

### CDx-Related Discussion in Scientific Advice Reports

**Figure 6:**
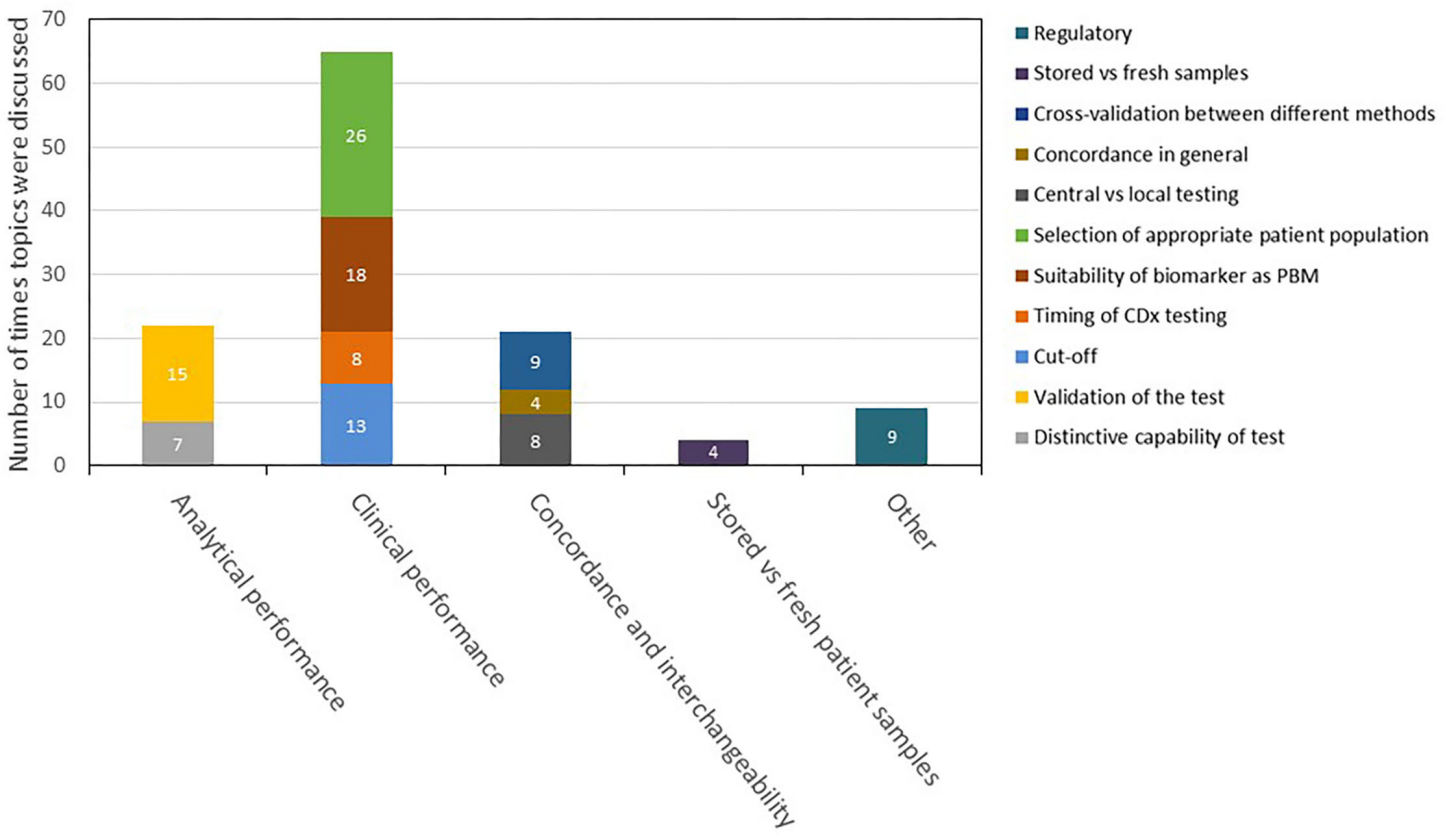
Description and categorization of remarks on CDx in scientific advice reports in 2016–2020. The four topics of analytical performance, clinical performance, concordance and interchangeability, and testing of stored vs. fresh patient samples were further divided into subtopics. Remarks that did not fit to any of the four topics were categorized as “other.” CDx, companion diagnostic.

For the 46 selected medicines, mentions of CDx analytical performance, clinical performance, testing of stored patient samples vs. fresh samples, and interchangeability including concordance testing and bridging studies in the scientific advice are summarized in a descriptive manner in Figure 6. Among all CDx-related discussions in these reports, 65 focused on the clinical performance of the CDx, 22 on the analytical performance, 21 on the concordance testing and bridging, and four on testing of stored vs. fresh patient samples.

### Percentage of Medicines With a CDx Compared to the Total of Marketing Authorizations in 2017–2019 and 2016–July 2020, Respectively

The relative number of medicines involving a CDx that were registered and mentioned in ARs in 2017–2019 is summarized in Table 2. Of the 167 registered medicines analyzed, 57 were registered in 2017 and 8.8% (five of 57) involved a CDx; 68 were registered in 2018 and 14.7% (10 of 68) involved a CDx; and 42 were registered in 2019 and 12% (five of 42) involved a CDx. In 2017 and 2018, all identified CDx were discussed in the ARs, whereas in 2018, this was the case for 80% (eight of 10) of the medicines. In the case of the EPARs for these medicines, these numbers were lower: 80% (four of five), 40% (four of 10), and 80% (four of five), respectively.

**Table 2:** The assessment of CDx in registered medicines at the EMA in 2017–2019.

**Year of authorization**	**Total number of medicines authorized**	**Number of medicines with CDx discussion in EPAR/total number of medicines with CDx authorized**	**Number of medicines with CDx discussion in AR/total number of medicines with CDx authorized**
2017	57	4/5 (80%)	5/5 (100%)
2018	68	4/10 (40%)	8/10 (80%)
2019	42	4/5 (80%)	5/5 (100%)

*AR, assessment report; CDx, companion diagnostic; EPAR, European public assessment report*.

### CDx Used in Clinical Studies for Registered Medicines

For half of the registered medicines with a CDx (10 of 20), a specific CDx brand was used in clinical trials, and these brands were mentioned in the SmPC or EPAR of these medicines. All 10 of these CDx tests—for example, AmoyDx™ for EGFR, Ventana anti-ALK (D5F3) for ALK, and the BRACAnalysis CDx^®^ for BRAC1—are those that are approved by the Food and Drug Administration. In Supplementary Table 3, the brand names of the CDx that were used in the clinical trials are mentioned.

For the other medicines (10 of 20) the information in the SmPC or EPAR on the type and brand of CDx used in the clinical trials was less extensive. An example is Rxulti (brexpiprazole). In the SmPC, it was stated that a genotyping essay was needed to establish the CYP2D6 metabolizer status, but the exact type of essay or the brand used in clinical trials was not specified (Supplementary Table 3).

## Discussion

In general, our study indicates that CDx accompanying a medicine are critically considered by the EMA in scientific advice provided to companies and during the registration process. Therefore, it can be stated that the EMA and the national agencies in EU member states have gained experience with critically assessing the CDx, as they will be formally required to do when the EU regulation on IVDs, Regulation (EU) 2017/746, becomes effective in 2022.

With respect to the pharmacological classes of medicines that were identified in our study, it is clear that CDx are mostly used in the oncological setting, with 85% and 92% of the CDx identified in the EPARs and scientific advice reports, respectively, belonging to this therapeutic class. This is in line with the known major involvement of specific molecular targets that are critical to be present in a patient's tumor for effective treatment by such oncological medicines. Therefore, it is understandable that oncological medicines represent the majority of medicines involving a CDx.

The relative number of medicines with a CDx involved remained relatively constant in 2017–2019, suggesting a stable proportion of medicines for which biomarker-based patient selection is relevant. At this stage, it is uncertain whether the proportion of medicines with a CDx involved will increase in the future. As such, the new IVDR is not specifically aiming to increase this proportion, however, it is in place to provide more robustness on the quality of the CDx that are marketed in the EU.

Four main topics are considered critical in relation to CDx and are discussed in many cases in scientific advice reports as well as during the registration process of a medicine encompassing a CDx. These can include 1) analytical performance of the CDx; 2) clinical performance of the CDx; 3) interchangeability of CDx assays, including concordance testing and bridging studies and tests for the same PBM; and 4) testing of stored or fresh patient samples, the latter encompassing both the appropriate preservation of the sample, e.g., fresh, frozen, or various fixations, as well the liquid biopsy or tumor biopsy. Of these topics, the clinical performance of the CDx is discussed most extensively both in the (public) ARs as well as in the scientific advice reports, followed by the analytical performance. In the ARs the category that had the most questions was “suitability of biomarker as PBM”. These findings are in line with a recent publication of Bakker et al. (19) in which the biomarker qualification procedure of the EMA was studied. They found that issues related to the rationale behind the suggested biomarker, as well as the suggested context of use were raised in >50% of the procedures.

When further examining EPARs and ARs, the discussions of CDx-related topics largely overlapped. In the standard registration process at the EMA, questions are often raised in the day 80 and day 120 ARs, which are responded to by the applicant in the course of the registration procedure. This was also apparent for CDx, and a significant number of specific CDx-related issues were discussed for the majority of medicines involving a CDx in the day 80 and day 120 ARs. In contrast, the EPAR is a more global summary of the assessment details for a medicine, focusing on the final outcome of the assessment. Therefore, information on the performance of the CDx in the EPAR provides somewhat less detail than the original ARs, since, in principle, only the conclusion of the discussion on CDx is provided in the EPAR. Furthermore, due to the known condensing effect in the process of establishing an EPAR (20, 21), if no major CDx-related issues were discussed during the registration process of the medicine, little to no information about the CDx may be provided in the EPAR. In fact, the EPAR should be read with the assumption in mind that if there is no information on the CDx in the EPAR, this means that no issues about the CDx were remaining at time of registration.

The types of CDx-related questions raised in scientific advice reports and during the registration process (as described in the ARs and EPARS) largely overlapped. However, regarding scientific advice, relatively more questions focused on the usefulness of a biomarker with a CDx for that biomarker which was not yet validated. Still, also in scientific advice, most CDx discussed were already analytically validated and, therefore, were developed to a reasonable extent for use during the clinical trials ongoing at the time of the scientific advice.

In the EU in general, no brand name of a CDx to be used for patient selection is indicated in the SmPC Sections 4.1–4.3. Instead, a statement is often included that an adequately validated CDx should be used. However, for transparency purposes and in order not to hinder the development of follow-on CDx, i.e., a CDx developed separately from the accompanying medicine, in many cases the brand name used in the clinical studies in support of registration of the medicine is indicated in Section 5.1 (pharmacodynamic properties) of the SmPC and is also mentioned in the EPAR. Of note, Food and Drug Administration-approved CDx are mentioned frequently. Transparency on the identity of used CDx in the SmPC or EPAR is not complete at this stage, with half of the SmPCs or EPARs (i.e., 10 out of 20) not providing such information, and improvement is needed. This is in line with recent observations published by Orellana Garcia et al. (9).

The strength of this research is the access of the authors to all regulatory information of the EMA, allowing an extensive overview of the aspects related to scientific advice and assessment of CDx. As a weakness, relevant assessment reports and scientific advice reports were retrieved by text search. It cannot be excluded that relevant procedures have been missed in case none of the keywords used was indicated in relation to the CDx. Still, we are confident that our results form a relevant representation of the experience at the EMA toward assessment of and scientific advice on CDx.

Overall, our study reveals that the EMA and the individual EU member states have gained relevant experience with critically assessing the CDx as part of registration procedures for medicines. This experience is expected to be of use in in the future, as formal consultation of the EMA or national medicines agencies regarding the performance and safety of a CDx will be required upon implementation of the EU regulation on IVDs, Regulation (EU) 2017/746, the first part related to new CDx becoming effective in 2022. In addition, the issues raised in the EPARs, ARs and scientific advice reports provide insight for applicants on aspects of CDx that need careful consideration.

## Conclusion

Our analysis—which is based on EU SmPCs, EMA EPARs, assessment reports, and scientific advice reports—indicates that, despite the absence of an obligation from a legal point of view, in recent years the EMA and the national agencies at the EU member states have gained experience regarding the assessment of CDx, most notably in relation to the analytical and clinical performance of the CDx. This experience may be useful and strengthens confidence about the future situation in which the EMA and national agencies of EU member states will be consulted formally by notified bodies regarding the performance and safety of CDx, leading to CE-marking of CDx in the process.

## Data Availability Statement

The data analyzed in this study is subject to the following licenses/restrictions: Part of the used reports are publicly available and links to these reports have been provided in the article. Access to the internal MEB assessment reports and EMA Scientific Advice database was granted to MM and AP due to their appointment at the Dutch Medicines Evaluation Board. Requests to access these datasets should be directed to AP, science@cbg-meb.nl.

## Author Contributions

MM and AP led the design. PN and GN led the data acquisition as well as analysis. Results were discussed among all four authors. All authors drafted the work, read and gave final approval of the version to be published, and provided substantial contributions to the interpretation of data for the work.

## Author Disclaimer

The views expressed in this article are the personal views of the authors and may not be understood or quoted as being made on behalf of or reflecting the position of the European Medicines Agency or one of its committees or working parties.

## Conflict of Interest

The authors declare that the research was conducted in the absence of any commercial or financial relationships that could be construed as a potential conflict of interest.

## Publisher's Note

All claims expressed in this article are solely those of the authors and do not necessarily represent those of their affiliated organizations, or those of the publisher, the editors and the reviewers. Any product that may be evaluated in this article, or claim that may be made by its manufacturer, is not guaranteed or endorsed by the publisher.

## Abbreviations

AR: (EMA) assessment report
CDx: companion diagnostics
CE mark: Conformitè Europëene mark
EMA: European Medicines Agency
EPAR: (EMA) European public assessment report
IVD: *in vitro* diagnostic
IVDR: EU regulation on *in vitro* diagnostics
PBM: predictive biomarker
Q&A: question and answer
SA: scientific advice
SAWP: (EMA) Scientific Advice Working Party
SmPC: summary of product characteristics.
