# Supplementary material for: Evaluation of Companion Diagnostics in Scientific Advice and Drug Marketing Authorization Applications by the European Medicines Agency
Source: Front Med (Lausanne). 2022 May 6;9:893028. doi: 10.3389/fmed.2022.893028 (PMC9120537; doi:10.3389/fmed.2022.893028)
Supplement: Supplementary file 1 [file Data_Sheet_1.pdf]

Supplementary Table 1. Information on Registered Medicines with Biomarkers Indicated in the SmPC or EPAR, Without Proposed CDx.

International non-proprietary name (INN)	EU medicine brand name	Biomarker	Purpose of biomarker (BM)	Specific test mentioned in SmPC or EPAR
allopurinol/lesinurad	Duzallo	HLA-B*5801	Complementary diagnostic for safety reasons in certain ethnic populations, not required	HLA-B*5801 genotyping assay
autologous CD34+ cells encoding βA-T87Q-globin gene	Zynteglo	TDT β0/β0 genotype	BM part of routine clinical assessment	-
azacitidine	Azacitidine Celgene	Marrow blasts	Part of complete blood count, clinical diagnosis	-
burosumab	Crysvita	Radiographic evidence of bone disease	BM serves clinical diagnosis	-
cerliponase alfa	Brineura	TPP1	BM serves clinical diagnosis	-
cladribine	Mavenclad	Lymphocyte count	BM serves routine safety assessment	-
darunavir/cobicistat/emtricitabine/tenofovir alafenamide	Symtuza	HIV-1 RNA copies and CD4+	BM part of routine clinical assessment	-
dolutegravir/lamivudine	Dovato	-	Absence of documented or suspected resistance to the integrase inhibitor class and to lamivudine serves clinical diagnosis	-
dolutegravir/rilpivirine	Juluca	NNRTI-associated mutations	BM may serve to clinically confirm absence of resistance	-
doravirine	Pifeltro	NNRTI-associated mutations	BM may serve to clinically confirm absence of resistance	-
doravirine/lamivudine/ enofovir disoproxil	Delstrigo	NNRTI-associated mutations	BM may serve to clinically confirm absence of resistance	-
dupilumab	Dupixent	Blood eosinophils and/or raised FeNO	BM serves clinical diagnosis type 2 inflammation	-
emicizumab	Hemlibra	Factor VIII inhibitors	BM serves clinical diagnosis	-
ertugliflozin	Steglatro	GFR	BM part of routine safety assessment	-
ertugliflozin/metformin hydrochloride	Segluromet	GFR	BM part of routine safety assessment	-
ertugliflozin/sitagliptin	Steglujan	GFR	BM part of routine safety assessment	-
fluciclovine (^{18}F)	Axumin	PSA	In vivo diagnostic	-

gemtuzumab ozogamicin	Mylotarg	CD33-positive	Complementary diagnosis, BM serves clinical diagnosis	-
glibenclamide	Amglidia	G6PD	Warning for G6PD carriers	-
ibalizumab	Trogarzo	HIV-1 RNA copies	BM part of routine clinical assessment	-
lutetium (^{177}Lu) oxodotreotide	Lutathera	Somatostatin receptor	In vivo diagnostic	-
metformin hydrochloride/ saxagliptin/dapagliflozin	Qtrilmet	GFR	BM part of routine safety assessment	-
mexiletine	Namuscla	CYP2D6	Complementary diagnosis, BM serves safety assessment	
niraparib	Zejula	BRCA mutation	BM not used for patient selection	-
ocrelizumab	Ocrevus	Imaging features characteristic of inflammatory activity	In vivo diagnosis	MRI
peramivir	Alpivab	H275Y mutation in Influenza A/H1N1 virus	Warning for reduced susceptibility	PCR
sofosbuvir/velpatasvir/ voxilaprevi	Vosevi	HCV genotype	BM part of routine clinical assessment	biochemical assay

Abbreviations: BM: biomarker, BRC: breast cancer suppressor gene, CD33: cluster of differentiation 33, CYP2D6: cytochrome P450 isoenzyme 2D6, FeNO: fraction of exhaled nitric oxide, G6PD: 6-phosphate dehydrogenase, GFR: glomerular filtration rate, HCV: hepatitis C virus, HIV-1: human immunodeficiency virus-1, MRI: magnetic resonance imaging, NNRTI: non-nucleoside reverse transcriptase inhibitor, PCR: polymerase chain reaction, PSA: prostate-specific antigen, TDT: transfusion-dependent β-thalassemia, TPP1: tripeptidyl-peptidase 1

Supplementary Table 2. Discussion on CDx Found in the EPARs for Products Registered at the EMA in 2017–2019

**Discussion of analytical performance**	
**Besponsa (inotuzumab ozogamicin)**	*1) Leukaemic cells were defined as CD22 positive if the level of fluorescence associated with binding of the CD22 monoclonal antibody [mouse anti-human monoclonal antibody clone RFB-4] was greater than the threshold value typically set based on the fluorescent intensity of leukemic cells stained with an isotype-matched control antibody.* ***<u>The validation report</u>*** *and addendums regarding peripheral blood specimens and inter-laboratory qualification* ***<u>were provided</u>****. (1)*<br><br>*2) Initially samples were considered to be positive if ≥20% of leukaemic blasts expressed surface CD22.* ***<u>The cut-off value was subsequently changed to >0% blasts</u>*** *(Protocol Amendment 2, StudyB1931022)* ***based on the finding that some subjects that failed screening in the Phase 1/2 study B1931010 tested CD22 negative locally*** *(≥20% cut-off)* ***but were CD22-positive based on Central laboratory testing.*** *(1)*
**Imfinzi (durvalumab)**	*The immuno-assays for quantification of durvalumab and soluble PD-L1 in human serum samples were* ***<u>adequately validated.</u>*** *(2)*
**Rubraca (rucaparib)**	*There was a high positive agreement between local and central BRCA results. The Clinical Trial Assay (as determined by a local laboratory) and CDx* ***<u>results appeared consistent</u>****. (3)*
**Rydapt (midostaurin)**<br><br>**(cross)validation**	*Test assay performance was regularly monitored (every 6 months) throughout the study at these participating central labs through the use of* ***<u>cross-validation panel testing</u>****. Laboratory failure resulted in suspension until CTA proficiency was demonstrated in the following round. (4)*
**Tecentriq (atezolizumab)**<br><br>**validation**	*Of note, although the immunohistochemistry assay is optimized to measure PD-L1 expression on both tumor cells (TCs) and tumour-infiltrating lymphocytes (ICs), the prevalence of PD-L1 expression on TCs in urothelial carcinoma is low. Therefore the VENTANA PD-L1 (SP142) IHC assay was* ***<u>not validated for intended use to measure PD-L1 expression on TCs in urothelial carcinoma</u>****. (5)*
**Vitrakvi (larotrectinib)**	*These techniques [Next Generation Sequencing (NGS) and Fluorescence in situ hybridization (FISH)]* ***<u>might give rise to false-</u>***

	positive *results as some NTRK gene rearrangements detected on DNA appear not to produce a fusion transcript. (6)*
**Discussion of clinical performance**	
**Alecensa (alectinib)**	*For a new ALKi to be used after failure on prior ALKi treatment it is clinically relevant to define whether resistance is related to secondary ALK mutations or other mechanisms underlying resistance; a priori activity e.g., in case of resistance due to activation of alternative signalling pathways bypassing ALK appears remote. Similarly, to what degree the secondary mutation affects the activity of the new compound is of importance. Such data are presently available to a very limited and non-informative extent.* ***The applicant is recommended to provide final analysis on secondary ALK mutation positive/negative samples and correlation with clinical outcome.*** *(7)*
**Imfinzi (durvalumab)**	*The study recruited all-comers with regard to PD-L1 expression, however, in the pre-defined subgroup analyses based on PD-L1 expression the applicant has applied a cut-off for PD-L1 expression at 25%. However, as discussed above****, it is not entirely clear how and why the cut-off is at 25%.*** *The Applicant has been asked to provide subgroup analyses in patients with <1%, >= 1%, 5% and 50%, and compare and discuss these results with the results based the on the 25% cut-off. (2)*
**Lorviqua (lorlatinib)**	*The approach used for ALK mutation RP2D selection is endorsed****. However, other relevant ALK-resistance mutations have been identified*** *for ceritinib (L1152R and F1174C/V) or alectinib (I1171N/T/S) [Muller et al. Onco Targets Ther. 2017; 10: 4535–4541] against which lorlatinib has not been tested in vitro using a PK/PD modelling-based approach. (8)*
**Mektovi (binimetinib)**	*In addition, genomic analysis of baseline samples remaining after centralized BRAF testing would be informative* ***to assess whether there is a relationship between baseline mutations and efficacy outcomes****. (9)*
**Rxulti (brexipiprazol)**	*The* ***necessity of a mandatory genotyping assay*** *should be discussed by the applicant. (10)*
**Rydapt (midostaurin)**	*The overall design of the study was considered adequate. The target patient population (patients with* ***≥5% FLT3-mutated alleles****), the comparator (standard induction and consolidation chemotherapy plus placebo), and the sequential dosing regimen of midostaurin were* ***considered acceptable*** *and have been previously agreed upon in an SAWP scientific advice (EMEA/H/SA/764/1/2006/PA/III). (4)*

**Talzenna (talazoparib)**	*A biomarker research program in blood and tumour, based on the EMBRACA study, is ongoing. The Applicant has initiated next generation-based DNA sequence analysis of tumour tissue samples collected from patients enrolled in the Phase 3 Study 673-301 (EMBRACA; C3441009). Results from this analysis will include BRCA1/2 tumour mutational status and the tumour mutational status of over 300 other genes. The applicant is recommended to **submit the biomarker report** containing the final results by 31 March 2020. (11)*
**Tecentriq (atezolizumab)**	*..which is partly attributed to the fact that PD-L1 expression proved to be **rather of prognostic than of predictive value** in this data set.*<br><br>*Finally, the applicant is recommended to provide a “biomarker analysis plan” with timelines and should submit the results of all ongoing and planned biomarker analyses post-approval. (5)*
**Vitrakvi (larotrectinib)**	*1) [An] expert expressed concern for the **very low percentage of breast cancers having proven NTRK fusions** as “oncogenic driver”. (6)*<br><br>*2) Several molecular tools are currently available for the detection of NTRK fusions in tumour specimens: Immunohistochemistry (IHC). **this tool must be validated against a sufficient cohort of NTRK-positive and –negative cases**. Data on sensitivity and specificity on all available essays should be made available. (6)*
**Xospata (gilteritinib)**	*Exploratory analysis based on **different FLT-3 internal tandem duplication (ITD) allelic ratio cut-off levels** have been provided, based on 335 patients that tested positive for FLT3 –ITD before inclusion in the study 2215-CL-0301. (12)*
**Discussion of interchangeability of assays (concordance testing and bridging studies)**	
**Besponsa (inotuzumab ozogamicin)**	*1) To assess **concordance between local and central testing**, patients were divided into groups (≥90%, ≥70-<90%, >0 -<70%, 0%)) based on the % of CD22+ leukaemic blasts in their baseline specimen. Similar results were seen for both ALL studies. There was **good concordance (~99.6%)** for evaluable subjects (252/253) between central and local testing for CD22-positivity > 0%. **However, concordance was poor (~37%)** between Central and Local laboratories with regards to the level of positivity (≥90%, ≥70 - <90%, 90%, >0 - <70%, 0%)). In general, a higher percentage of blasts were CD22-positive when measured by central versus local laboratory testing, suggesting that the central laboratory test was more sensitive.*

	There was reasonable concordance (87.7%) between bone marrow and peripheral blood by Central laboratory for assessment of the % blasts that were CD22 + at baseline based on 57 evaluable patients.<br><br>*"The various local tests used in the two ALL clinical studies to screen for CD22 at baseline were not as sensitive as the single central laboratory test." Of these 367 patients, only one (who subsequently exhibited 86% CD22 positivity by central laboratory test) had CD22-negative B-cell ALL by local lab testing (ie, CD22 positivity=0%), consistent with CD22 positivity being an inclusion criterion for these studies.* ***It is not possible to determine the true proportion of locally tested patients who were CD22-negative****, since local laboratory CD22 test results were not formally captured for patients who failed screening and not all those who were screened negative locally were sent for central review. (1)*<br><br>*2)* ***The validation report*** *and addendums regarding peripheral blood specimens and* ***inter-laboratory qualification*** ***were provided.*** *(1)*
**Rubraca (rucaparib)**	*1) Central testing in the clinical studies was initially performed using the clinical trial assay (CTA) and subsequently* ***bridged*** *to the CDx. The CTA (as determined by a local laboratory) and CDx* ***results appeared consistent.***<br><br>*2) Tumour specimens from 79 patients in the primary efficacy (PE) population were received for central BRCA mutation testing at Foundation Medicine Inc. There was a* ***high positive agreement between local and central BRCA results****. (3)*
**Talzenna (talazoparib)**	*There are methods available in a research setting, to test for BRCA1/2 locus-specific loss of heterozygosity; however,* ***the Scientific Advisory Group (SAG) could not confirm to what extent any particular test is well-established.*** *Furthermore, the relationship between loss of heterozygosity (LOH) and Homologous recombination deficiency (HRD) in germline mutation-associated and sporadic breast tumours is unclear, and mechanisms apart from LOH do operate in gBRCA-associated breast cancer as a mechanism for biallelic inactivation of BRCA1 and BRCA2, so* ***the clinical utility of such tests [HRD testing] over BRCA testing is not likely to be important in the context of treatment effect with PARP inhibition.*** *(11)*
**Vitrakvi (larotrectinib)**	*However, given the multitude of 5' partners involved in NTRK1/2/3 fusion genes, assays that allow for the detection of multiple variants in a single test, including NGS-based RNA and DNA approaches, have been widely used in large academic centres.* ***The adoption of these NGS-based methods seems to be the better option despite that testing can also be performed with immunohistochemistry followed by confirmatory NGS****. (6)*
**Discussion of testing stored- vs fresh patient samples**	

**Rubraca (rucaparib)**	*Local BRCA testing of DNA extracted from blood or buccal samples. Archival formalin-fixed paraffin-embedded tumour tissue samples were **retrospectively requested** to confirm the presence of BRCA mutations utilizing the FMI [foundation Medicine, Inc] validated tests (CTA then CDx). (3)*
**Vitrakvi (larotrectinib)**	*1) Liquid biopsies should be further investigated to address this challenge. These [liquid biopsies] should be performed in projects with a joint tumour biopsy **up-front** and ideally at cancer progression. For the rare indications listed above, such **studies should be conducted to refine the understanding on patient selection**. (6)*<br><br>*2) CHMP questions with SAG answers: During the initial discussion, one expert raised the possibility to have a **tissue independent approval for cancers with proven NTRK fusions** as oncogenic "drivers", provided that next generation sequencing (NGS) could exclude other alterations being significant drivers for tumour progression. However, **data are lacking to establish the efficacy of such possible strategy**.*<br><br>***A main drawback of the studies presented was the lack of comprehensive sequencing (NGS) of relevant tumour tissue reflecting the tumour characteristics at the time of treatment with larotrectinib**. Such studies are necessary to understand the role of NTRK fusions in the context of other disease characteristics to allow patient selection and further evidence-based development in other indications or across groups of tumour types. Although the burden associated with biopsies is well-recognised most patients understand its importance and are willing to undergo the procedure. (6)*

References Supplementary Table 2

1. Besponsa EPAR, Public Assessment Report, procedure no. EMEA/H/C/004119/0000. https://www.ema.europa.eu/en/documents/assessment-report/besponsa-epar-public-assessment-report_en.pdf. Accessed Jun 11, 2020.

2. Imfinzi EPAR, Public Assessment Report, procedure no. EMEA/H/C/004771/0000. https://www.ema.europa.eu/en/documents/assessment-report/imfinzi-epar-public-assessment-report_en.pdf. Accessed Jun 11, 2020.

3. Rubraca EPAR, Public Assessment Report, procedure no. EMEA/H/C/004272/0000. https://www.ema.europa.eu/en/documents/assessment-report/rubraca-epar-public-assessment-report_en.pdf. Accessed Jun 11, 2020.

4. Rydapt EPAR, Public Assessment Report, procedure no. EMEA/H/C/004095/0000. https://www.ema.europa.eu/en/documents/assessment-report/rydapt-epar-public-assessment-report_en.pdf. Accessed Jun 11, 2020.

5. Tecentriq EPAR, Public Assessment Report, procedure no. EMEA/H/C/004143/0000. https://www.ema.europa.eu/en/documents/assessment-report/tecentriq-epar-public-assessment-report_en.pdf. Accessed Jun 11, 2020.

6. Vitrakvi EPAR, Public Assessment Report, procedure no. EMEA/H/C/004919/0000. https://www.ema.europa.eu/en/documents/assessment-report/vitrakvi-epar-public-assessment-report_en.pdf. Accessed Jun 11, 2020.

7. Alecensa EPAR, Public Assessment Report, procedure no. EMEA/H/C/004164/0000. https://www.ema.europa.eu/en/documents/assessment-report/alecensa-epar-public-assessment-report_en.pdf. Accessed Jun 11, 2020.

8. Lorviqua EPAR, Public Assessment Report, procedure no. EMEA/H/C/004646/0000. https://www.ema.europa.eu/en/documents/assessment-report/lorviqua-epar-public-assessment-report_en.pdf. Accessed Jun 11, 2020.

9. Mektovi EPAR, Public Assessment Report, procedure no. EMEA/H/C/004579/0000. https://www.ema.europa.eu/en/documents/assessment-report/mektovi-epar-public-assessment-report_en.pdf. Accessed Jun 11, 2020.

10. Rxulti EPAR, Public Assessment Report, procedure no. EMEA/H/C/003841/0000. https://www.ema.europa.eu/en/documents/assessment-report/rxulti-epar-public-assessment-report_en.pdf. Accessed Jun 11, 2020.

11. Talzenna EPAR, Public Assessment Report, procedure no. EMEA/H/C/004674. https://www.ema.europa.eu/en/documents/assessment-report/talzenna-epar-public-assessment-report_en.pdf. Accessed Jun 11, 2020.

12. Xospata EPAR, Public Assessment Report, procedure no. EMEA/H/C/004752/0000. https://www.ema.europa.eu/en/documents/assessment-report/xospata-epar-public-assessment-report_en.pdf. Accessed Jun 11, 2020.

Supplementary Table 3. Information on Registered Medicines With Companion Diagnostics, With the Brand Names of the CDx That Were Used in the Clinical Trials

Information taken from the SmPC or EPAR

International non-proprietary name (INN)	EU medicine brand name	Biomarker	CDx brand used in clinical studies, as indicated in the SmPC or EPAR
alectinib	Alecensa	ALK	Ventana anti-ALK (D5F3) (IHC)
brigatinib	Alunbrig	ALK	Vysis ® Break Apart FISH Probe Kit<br>FoundationOne NGS platform
lorlatinib	Lorviqua	ALK	Vysis ® Break Apart FISH Probe Kit<br>Ventana ALK (D5F3) CDx Assay)
binimetinib	Mektovi	BRAF V600 mutation	n.m.
encorafenib	Braftovi	BRAF V600 mutation	n.m.
rucaparib	Rubraca	BRCA1/2 mutations	n.m.
talazoparib	Talzenna	BRCA1/2 mutations	Myriad BRACAnalysis test (CLIA and QSR/CDx versions)
dacomitinib	Vizimpro	EGFR activating mutations	Qiagen therascreen EGFR Mutations Detection Kit<br>AmoyDxTM EGFR Mutations Detection Kit
gilteritinib	Xospata	FLT3 mutation	LeukoStrat® CDx FLT3 Mutation Assay
midostaurin	Rydapt	FLT3 mutation	n.m.
atezolizumab	Tecentriq	PD-L1	Ventana PD-L1 (SP142) Assay
durvalumab	Imfinzi	PD-L1	Ventana PD-L1 (SP263) IHC assay
inotuzumab ozogamicin	Besponsa	CD22	n.m.
larotrectinib	Vitrakvi	NTRK gene fusion	NGS testing methods were used on DNA (MSK-IMPACT, FoundationOne, OncoPlex), RNA (Archer FusionPlex, OmniSeq comprehensive, Thermo Fisher Oncomine Focus), on circulating tumor DNA (Guardant360)

abemaciclib	Verzenios	HER2 negative	Other, according to ASCO guidelines
neratinib	Nerlynx	HER2 positive	PathVysion HER2 Kit (FISH)
ribociclib	Kisqali	HER2 negative	n.m.
brexpiprazole	Rxulti	CYP2D6 PM	n.m.
voretigene neparvovec	Luxturna	RPE65 mutations	n.m.
tezacaftor / ivacaftor	Symkevi	Homozygous for the F508del mutation or heterozygous + one of the mutations mentioned in the SmPC	n.m.

Abbreviations: ALK: anaplastic lymphoma kinase, BRAF: B-raf proto-oncogene, BRCA: breast cancer gene, CD22: cluster of differentiation-22, CYP2D6: cytochrome P450 isoenzyme 2D6, EGFR: epidermal growth factor receptor, FISH: fluorescence in situ hybridization, FLT3: fms-like tyrosine kinase 3, HER: human epidermal growth factor receptor, IHC: immunohistochemistry, NGS: next generation sequencing, n.m.: not mentioned, NTRK: neurotrophic tyrosine kinase receptor, PD-L1: programmed death-ligand 1, RPE65: retinal pigment epithelium-specific 65 kDa protein
